# HAEMFIX: Impact of Switching From SHL‐FIX to EHL‐FIX in Patients With Haemophilia B

**DOI:** 10.1111/hae.70157

**Published:** 2025-10-25

**Authors:** Jasmin Lonardi, Susan Halimeh, Sylvia von Mackensen, Lisa Kleinlein, Juliet Fleischer, Henri Funk, Julia Hölz, Johannes Holzapfel, Sabrina Juranek, Victoria Lieftüchter, Christoph Bidlingmaier, Martin Olivieri

**Affiliations:** ^1^ Paediatric Thrombosis and Haemostasis Unit Paediatric Haemophilia Centre Dr. Von Hauner Children´s Hospital LMU Clinic Munich Germany; ^2^ Coagulation and Thrombosis Centre (GZRR) Duisburg Germany; ^3^ Department of Medical Psychology University Medical Centre Hamburg‐Eppendorf Hamburg Germany; ^4^ Statistical Consulting Unit StaBLab LMU Munich Munich Germany; ^5^ Statistical Consulting Unit StaBLab Department of Geography LMU Munich Munich Centre for Machine Learning (MCML) Munich Germany

**Keywords:** annual bleeding rate, extended half‐life factor IX, haemophilia B, health‐related quality of life, standard half‐life factor IX, therapy switching

## Abstract

**Introduction:**

Haemophilia B is an X‐linked recessive bleeding disorder caused by coagulation factor IX (FIX) deficiency. Treatment involves intravenous replacement of FIX. Recently, extended half‐life (EHL) FIX products have been introduced alongside standard half‐life (SHL) products to optimize therapy.

**Aim:**

This study evaluated bleeding rates, joint health, factor consumption, dosage, and health‐related quality of life (HRQoL) in patients switching from SHL‐ to EHL‐FIX products, as well as in those exclusively treated with EHL‐FIX.

**Methods:**

Retrospective data from the medical records of 37 children with haemophilia B treated between 2010 and 2023 at two German Haemophilia Care Centres were analysed. HRQoL was assessed cross‐sectionally using haemophilia‐specific and generic questionnaires.

**Results:**

Twenty‐seven patients (median age: 12 years, range 2–19 years) switched from SHL‐ to EHL‐FIX, while 10 received EHL‐FIX from the start of prophylaxis. The mean annual bleeding rate (ABR) improved from 6.01 ± 7.01 (SHL) to 2.85 ± 3.42 (EHL). Factor consumption (159,577.8 ± 99,817.9 IU/year), dosage (118.9 ± 50.3 IU/kg/week) and infusion frequency (145 ± 35.6 infusions/year) decreased after switching (100,247.7 ± 46,268.6 IU/year; 56.4 ± 23.7 IU/kg/week; 55.1 ± 9.8 infusions/year). HRQoL improved in both self‐reports and parent reports. No severe adverse events occurred.

**Conclusion:**

Switching from SHL‐FIX to EHL‐FIX in children with haemophilia B is safe and may improve outcomes by reducing bleeding rates, infusion frequency, and factor consumption while enhancing joint health and HRQoL.

## 1 Introduction

Haemophilia B (HB) is a hereditary X‐linked bleeding disorder characterized by reduced factor IX (FIX) levels [1]. The estimated prevalence of haemophilia by the World Federation of Haemophilia (WFH) is 400,000 people worldwide. HB is less common, with an incidence of 1:30,000 [2, 3]. The severity of the bleeding tendency correlates with the measured residual factor concentration and is classified as mild (>5%–40%), moderate (1%–5%) or severe (<1%) [1]. Recurrent bleeding into joints and muscles causes long‐term musculoskeletal impairment, leading to chronic pain, haemophilic arthropathy and disability. Soft tissue and intracranial bleeding are rare but can cause permanent damage to internal organs and might be life‐threatening [4]. To avoid these recurrent spontaneous bleeds, early start of prophylaxis by intravenous substitution of FIX is recommended [5, 6]. To achieve sufficient trough levels, standard half‐life (SHL) FIX products must be administered up to 2 or 3 times a week. In accordance with the recommendation of the WFH, the factor trough level should be 3%–5% [7]. The development and licensing of extended half‐life (EHL) factor concentrates represents a significant improvement in the treatment of HB. In different studies, EHL‐FIX products are reported to result in longer half‐lives, higher trough factor levels and lower infusion frequencies than SHL‐FIX products do [8, 9].

In Germany, different EHL‐FIX products are licenced for use in children, previously untreated patients (PUP) and previously treated patients (PTP): Albutrepenonacog alfa, rFIX‐FP (Idelvion)—recombinant fusion protein from FIX and albumin [10, 11], Eftrenonacog alfa, rFIX‐Fc (Alprolix)—fusion of FIX with the Fc domain of immunoglobulin G1 (IgG1) [12, 13] and nonacog beta pegol, PEG‐rFIX (Refixia)—with the coagulation factor covalently bound to polyethylene glycol (PEG) [14]. Since the approval of these products, patients have been switched to them consecutively.

The aim of this study was to evaluate the real‐world impact of paediatric patients switching from SHL‐ to EHL‐FIX products on clinical endpoints such as bleeding rates, joint health, factor consumption, dosage and health‐related quality of life (HRQoL).

## 2 Materials and Methods

Patient data for this combined retrospective‐analytical cross‐sectional study were collected from two Haemophilia Comprehensive Care Centres at Dr. von Hauner Children's Hospital and at the Rhein‐Ruhr Coagulation Centre. A total of 37 patients were included.

Patient data from between 2010 and 2023 were obtained from medical charts, paper patient files and electronic patient records (Haemoassist) and were analysed retrospectively. All study participants were regularly treated in the respective centres during the observed period, and informed consent was obtained from all patients and/or parents. No patient was excluded during the study. The time of study enrolment was from 09/2022 to 09/2023.

The total observation time per patient was chosen according to the time after switching until 2022, up to a maximum of 12 years (6 years before switching and 6 years after switching) for patients who switched after licensing in 2016. Furthermore, patients were divided into four different age groups, 0–2, 3–6, 7–12 and 13–18 years, for a more detailed analysis. This grouping was based on the dynamic age of the individual study participants, as retrospective longitudinal data were collected. With this method, a participant can be assigned to several age groups, and all their data can be included in the analysis.

Factor consumption and infusion frequency were recorded based on the batch documentation (electronically via Haemoassist or via paper form) made by either the patient or their caregivers and reported in 1‐year intervals. Annual bleeding rates were collected using data documented by patients or parents, considering location and type (spontaneous or traumatic). Dosing was determined individually depending on clinical bleeding tendency, trough levels and according to the suitable pack size.

For each study participant we retrospectively collected real FIX trough levels, measured on different days after the last substitution, and obtained an estimated value of each patients’ trough level 7 days after substitution. The trough levels and half‐lives of rFIX‐FP, rFIX‐Fc and PEG‐rFIX were calculated using the WAPPS‐Hemo platform. Individually generated pharmacokinetic profiles were used to generate estimated values for trough levels and half‐lives. In this study, the balanced estimate was used [15].

The Haemophilia Joint Health Score (HJHS) was used to assess joint health in the participant group at study enrolment. However, for the primary purposes of this study, we report only the most recent HJHS from the year prior to study enrolment [16].

The HRQoL of all patients under treatment with EHL‐FIX was assessed at the time of enrolment in the study via standardized and validated generic (EQ‐5D‐Y‐3L, EQ‐5D‐3L [17, 18]) and haemophilia‐specific (Haemo‐QoL SF, Haem‐A‐QoL [19, 20]) questionnaires. The EQ‐5D‐Y‐3L is available for children and adolescents aged 8–10 years and consists of five domains (‘Mobility’, ‘Self‐Care’, ‘Usual activities’, ‘Pain/Discomfort’ and Anxiety/Depression’) with three answer categories ranging from ‘no/not’, ‘some/a bit’ and ‘a lot of problems/very’, with high values indicating high impairments in health status. The visual analogue scale of the EQ‐5D (EQ VAS) ranges from 0 (worst imaginable health) to 100 (best imaginable health). The Haemo‐QoL SF is available as self‐reports and parents’ proxy reports for two age groups (4–7 years: interview form with 16 items and three answer categories ranging from ‘never’, ‘sometimes’ and ‘always’; 8–17 years: self‐rated form with 35 items and five answer categories ranging from ‘never’, ‘rarely’, ‘sometimes’, ‘often’ and ‘always’), with high values indicating high impairments in HRQoL. Children younger than 4 years did not complete the Haemo‐QoL SF questionnaire.

Patients who were previously treated with SHL‐FIX were asked to estimate their HRQoL while using SHL‐FIX retrospectively regardless of the time interval between switching and study enrolment. The questionnaires used to retrospectively assess HRQoL (the Haemo‐QoL Index [21] and the Haem‐A‐QoL) were adapted accordingly for this study. In the Haemo‐QoL Index, the time frame was changed from ‘in the last 4 weeks’ to ‘before switching to the new medication’. Regarding the Haem‐A‐QoL, one question was selected for each of the 10 dimensions, with the time frame being changed from ‘in the last 4 weeks’ to ‘before switching to the new medication’.

### 2.1 Statistical Analyses

All the data are presented descriptively as the means ± standard deviations, medians and ranges (minimum‒maximum) or numbers and frequencies according to their distributions. To test for significant differences in the effects of EHL‐FIX and SHL‐FIX, bleeding counts, factor consumption rates and infusion frequencies were modelled using generalized linear models. We include annual and ongoing temporal effects, and the centre as fixed effects in each model. To account for individual characteristics of the patients each model also includes a random effect per patient. *p* values < 0.05 were considered significant. The spontaneous, traumatic and total bleeding counts were modelled using the Poisson distribution assumption and log‐link. The logarithmic consumption and infusions were modelled using the assumption of a normal distribution and identity link. The models were selected using the R package ‘DHARMa’ and its simulateResiduals function.

## 3 Results

**FIGURE 1:**
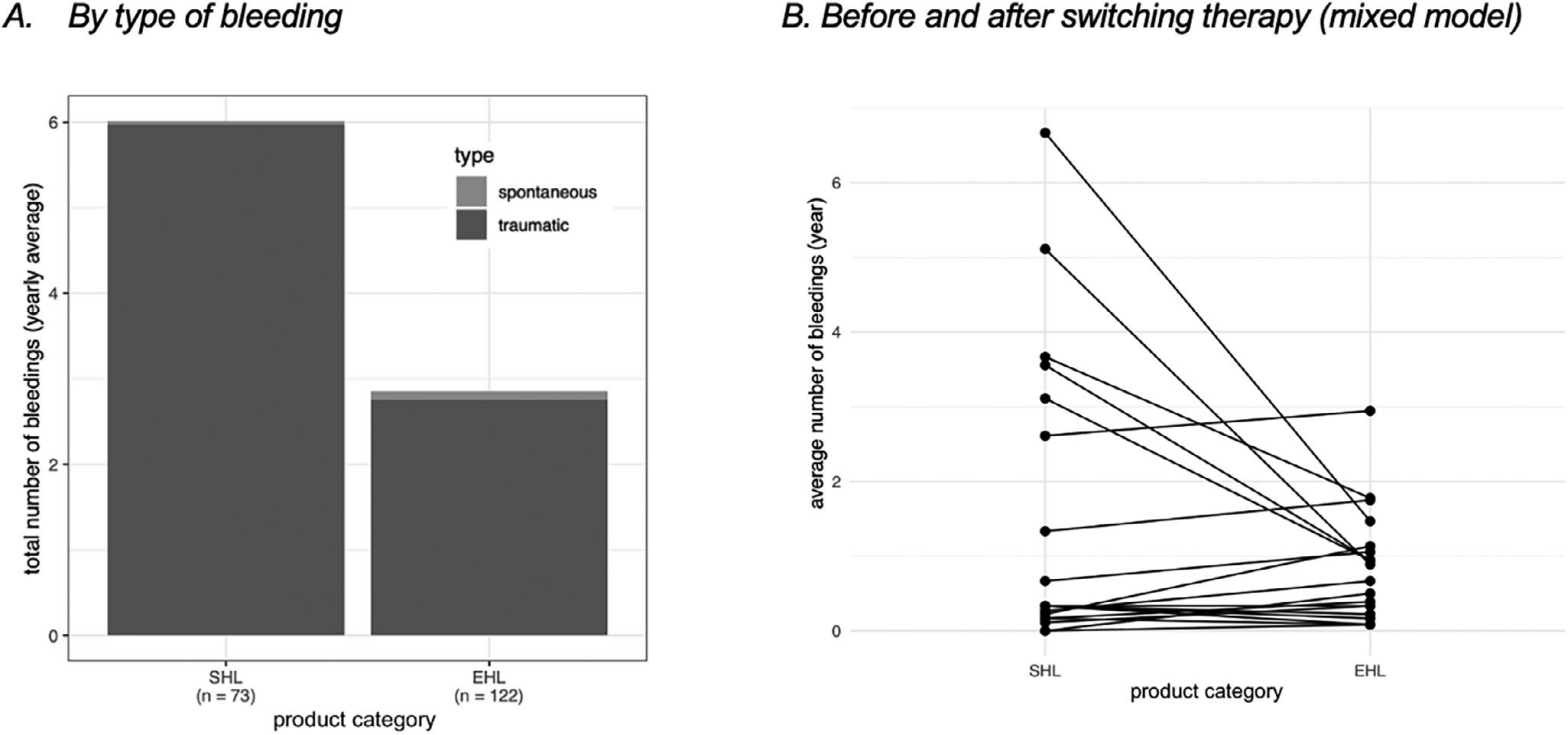
Number of annual total bleeds. (A) Mean annual bleeding rates categorized by type (spontaneous, traumatic) and product category (SHL, EHL) (*n* = 195). (B) Individual changes in annual bleeding rates per patient following therapy switch from SHL to EHL. Each point represents an individual patient's average annual bleeding frequency (*n* = 19).

**FIGURE 2:**
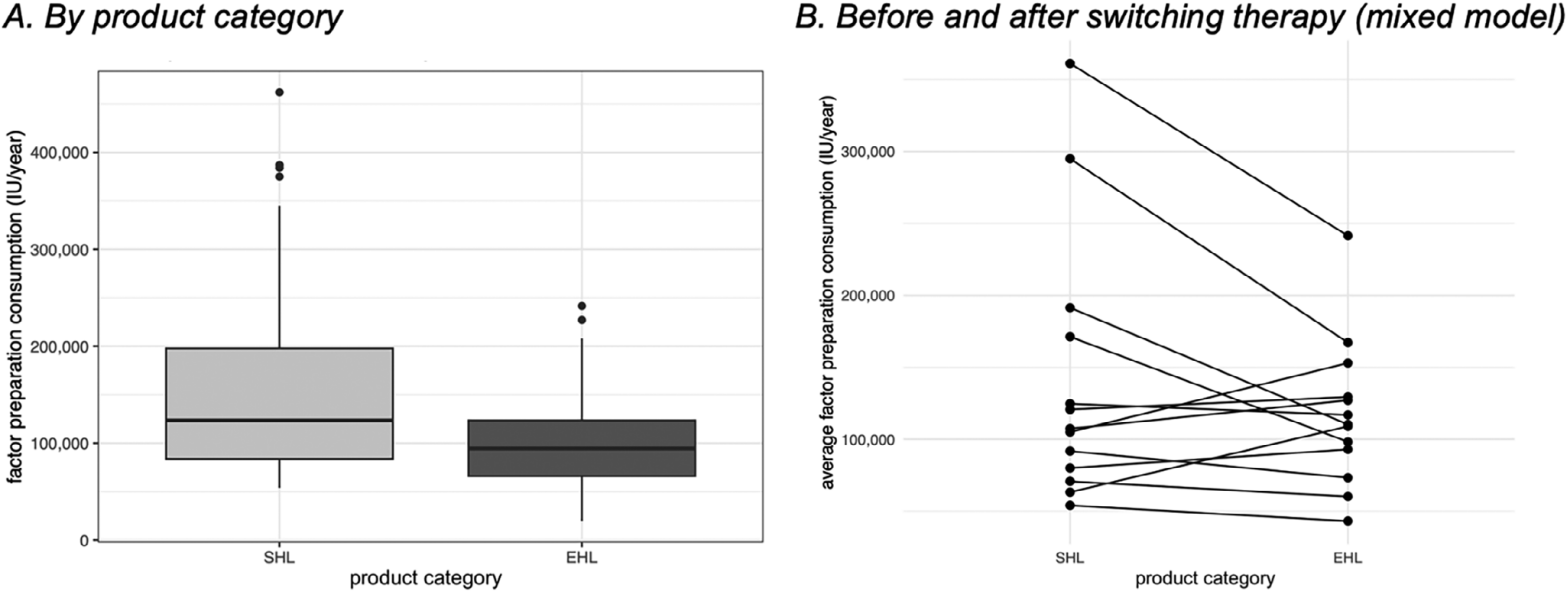
Annualized factor consumption. (A) Annualized factor consumption (IU/year) by product category (SHL, EHL) (*n* = 54). (B) Individual changes in annualized factor consumption per patient before (SHL) and after switching therapy to EHL. Each line represents an individual patient's change in consumption (*n* = 13).

**FIGURE 3:**
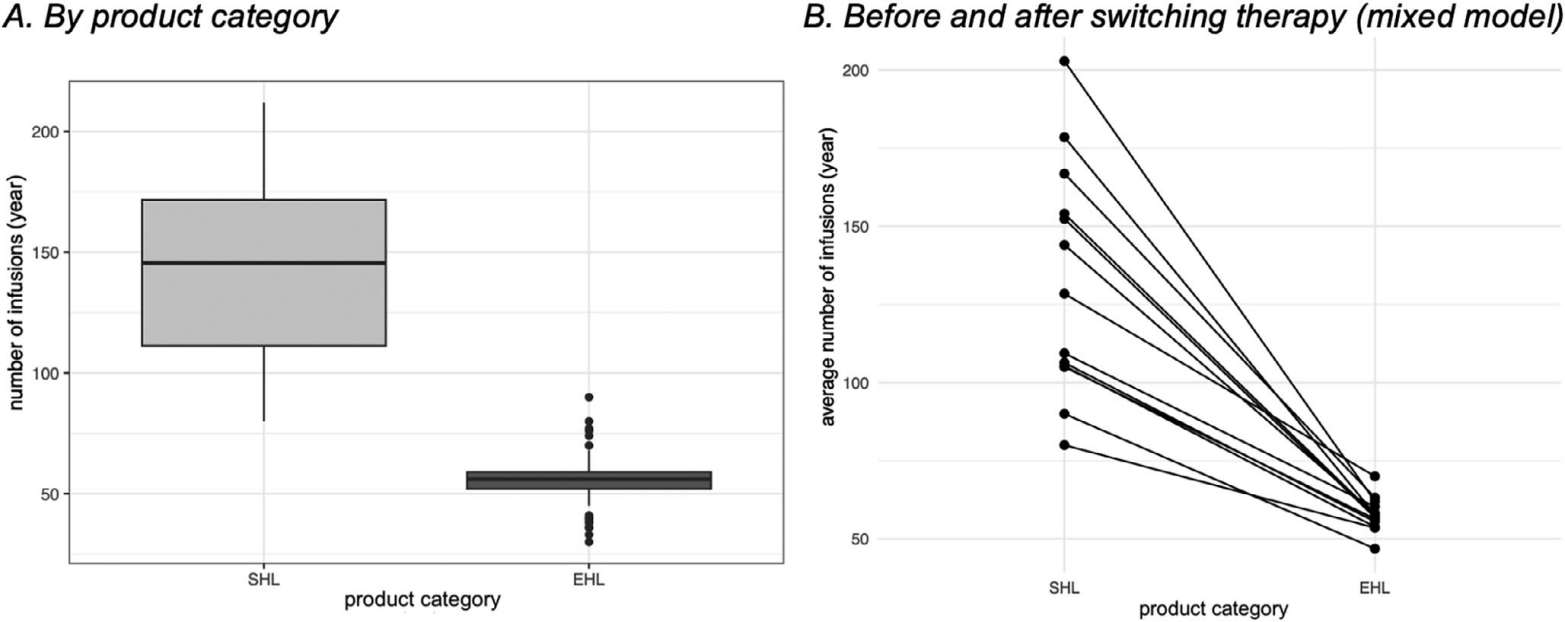
Number of annualized infusions. (A) Annualized infusion frequency (number of infusions/year) by product category (SHL, EHL) (*n* = 54). (B) Individual patient trajectories illustrating the change in annual infusion frequency before (SHL) and after switching to EHL therapy. Each line represents one patient's infusion frequency change (*n* = 13).

**FIGURE 4:**
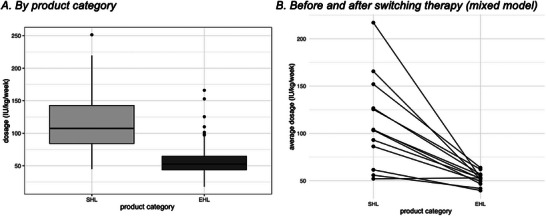
Dosage per IU/week. (A) Weekly dosage (IU/kg/week) by product category (SHL, EHL) (*n* = 54). (B) Individual patient trajectories showing the change in weekly dosage following therapy switch from SHL to EHL. Each line represents the dosage change of one patient (*n* = 13).

As shown in Table 1, 37 male paediatric patients with HB were included in the study. The median age was 12 years (range 2–19 years). Twenty‐six (70%) patients were diagnosed with severe, two (5%) with moderate and nine (24%) with mild haemophilia. Eighty‐six percent (*n* = 32) of patients received prophylactic treatment, and 14% (*n* = 5) received on‐demand treatment with FIX. At the time of data collection, all patients on prophylaxis used EHL‐FIX concentrates. Among the patients on prophylaxis, 19 patients were treated with rFIX‐FP, 10 with rFIXFc and 3 with PEG‐rFIX. The patients who received on‐demand treatment received rFIXFc (*n* = 4) and rFIX‐FP (*n* = 1). The previous use of SHL‐FIX preparations was recorded for 27 patients, and the products used were Nonacog alfa (Benefix, *n* = 15), plasmatic coagulation factors (Octanine (*n* = 6), Immunine (*n* = 3), Haemonine (*n* = 2)) and Nonacog gamma (Rixubis, *n* = 1). Individual comparison was made for all patients on prophylactic treatment and after switching (*n* = 22). Figures 1, 2, 3B and 4B shows patients with complete data set. For the prophylactic treatment with EHL‐FIX, the mean observation period was 4.0 ± 1.72 years (*n* = 32). Under SHL‐FIX therapy, the mean observation period was 3.68 ± 2.01 years (*n* = 22).

**TABLE 1:** Clinical characteristics of patients (*n* = 37) categorized by haemophilia severity.

	Total (*n* = 37)		Severe Haemophilia (*n* = 26)		Moderate Haemophilia (*n* = 2)		Mild Haemophilia (*n* = 9)	
	*N*	%	*N*	%	*N*	%	*N*	%
Age group								
2–7 years	13	35.1	10	38.5	1	50	2	22.2
8–16 years	18	48.6	11	42.3	0	0	7	77.8
≥17 years	6	16.2	5	19.2	1	50	0	0
Therapy regime								
Prophylaxis	32	86.5	26	100	2	100	4	44.4
On‐demand	5	13.5	0	0	0	0	5	55.6
EHL‐FIX product								
rFIX‐FP	20	54.1	18	69.2	1	50	1	11.1
rFIXFc	14	37.8	5	19.2	1	50	8	88.9
PEG‐rFIX	3	8.1	3	11.5	0	0	0	0
Switch in therapy								
From SHL‐ to EHL‐FIX product	27	73.0	22	84.6	1	50	5	55.6

*Note*: *n* = number of patients; % = percentage within each severity group.

Under prophylaxis with SHL‐FIX (*n* = 32), the overall mean ± SD annualized bleeding rate (ABR) was 6.01 ± 7.01, the mean traumatic annualized bleeding rate (tABR) was 5.97 ± 7.02, and the mean spontaneous annualized bleeding rate (sABR) was 0.04 ± 0.2, whereas for EHL‐FIX, the overall ABR was 2.85 ± 3.42, the tABR was 2.75 ± 3.33 and the sABR was 0.1 ± 0.37 (see Figure 1A). The proportion of patients with zero bleeds was 73.5% for SHL‐FIX and 72.4% for EHL‐FIX. The percentage of patients with an ABR of 1–2 was 10% and an ABR of >2 was found in 16.5% for SHL‐FIX. ForEHL‐FIX ABR was 1–2 in 13.4% and ABR was >2 in 14.2%. The mixed model showed that when EHL‐FIX was used instead of SHL‐FIX, the total number of expected bleeds decreased by a factor of 0.648 if all other variables remained constant (*p* = 0.01) (see Figure 1B). The annual bleeding rates of patients treated on prophylaxis by age group are shown in Table 2. The mean ± SD number of joint bleeding events per year was 1.5 ± 1.54 with SHL‐FIX therapy. Joints were the most common site of bleeding (1.1 ± 1.44 per year), accounting for 37% of all recorded bleeding events with EHL‐FIX therapy (data not shown here).

**TABLE 2:** Annualized bleeding rates (ABR) for traumatic (tABR) and spontaneous (sABR) bleeds by age group and product category.

Prophylaxis	Age group	tABR		sABR	
Product category	Years	Mean	SD	Mean	SD
SHL	0–2	10.86	5.83	0.07	0.27
	3–6	7.18	7.62	0.03	0.16
	7–12	0.55	0.61	0.05	0.22
EHL	0–2	3.81	5.4	0.0	0.0
	3–6	2.73	2.39	0.09	0.43
	7–12	2.88	3.13	0.12	0.39
	13–18	1.87	2.11	0.13	0.43

The mean ± SD annual factor consumption was greater in patients using SHL‐FIX (159,577.8 ± 99,818 IU/year) and decreased after switching to EHL‐FIX (100,247.7 ± 46,269 IU/year) (see Figure 2A). This effect was constant across the different age groups despite an increase in factor consumption in the older groups: 0–2 years, SHL‐FIX 132,189 ± 65,873 IU versus EHL‐FIX 58,900 ± 28,172 IU; 3–6 years, SHL‐FIX 185,505 ± 117,605 IU versus EHL‐FIX 71,750 ± 22,787 IU; 7–12 years, SHL‐FIX 112,489 ± 48,832 IU versus EHL‐FIX 96,702 ± 29,131 IU; 13–18 years, SHL‐FIX 189,000 ± NA (NA: not applicable) IU versus EHL‐FIX 152,018 ± 43,359 IU. The mixed model showed that when EHL‐FIX was used rather than SHL‐FIX, the expected consumption could be reduced significantly by a factor of 0.474, with all other variables held constant (*p* = 0.001) (see Figure 2B). In two patients we observed an increase in factor consumption despite switching to EHL. In one patient the reason for this dose escalation was haemophilic arthropathy. In the second patient no reason was documented.

The mean ± SD number of intravenous infusions per year for patients receiving SHL‐FIX was 145.0 ± 35.6. For patients receiving EHL‐FIX, the mean was 55.1 ± 9.8/year (see Figure 3A). The mixed model analysis revealed that the use of EHL‐FIX reduced the number of infusions by a factor of 0.423 (*p* = 0.001), with all other variables held constant (see Figure 3B). The extended half‐life of rFIX‐FP, rFIXFc and PEG‐rFIX led to a reduction in the infusion frequency to once a week in 88% of patients, with 9% requiring infusions every 10 or 14 days.

The mean ± SD dose of SHL‐FIX was 118.9 ± 50.3 IU/kg/week. The mean dose for therapy with EHL‐FIX was 56.4 ± 23.7 IU/kg/week (see Figure 4A). If there was dosage given (dosage >0), the mixed model analysis indicated that the utilization of EHL‐FIX resulted in a reduction of the dosage by 0.490 times (*p *= 0.001) holding all other variables constant (see Figure 4B).

The mean ± SD balanced estimated half‐life of the EHL‐FIX products was 146.58 ± 54.05 h. rFIX‐FP had a terminal half‐life (THL) of 143.62 h, rFIXFc had a THL of 158.97 h, and PEG‐rFIX had a THL of 130.34 h. The mean plasma level of the EHL‐FIX preparations 7 days after infusion was 0.13 ± 0.07 IU/mL. Using the WappsHemo tool, the estimated trough level 7 days after infusion was 0.16 IU/mL with rFIX‐FP, 0.08 IU/mL with rFIXFc and 0.14 IU/mL with PEG‐rFIX (data not shown here).

The HJHS was determined in 33 study participants (25 severe, 1 moderate, 7 mild) within the previous year, resulting in a mean HJHS of 0.88 (median 0, range 9). Haemophilic arthropathy was present in two patients with severe haemophilia. The occurrence of a target joint was described in one patient and was localized in the right elbow.

The HRQoL questionnaires were completed by 30 of the 37 participants. Two patients did not complete the Haemo‐QoL questionnaire, which is only available for children ≥4 years, because their age was <4 years. In the 4–7‐year age group, the mean ± SD Haemo‐QoL SF I total score was higher for the parents’ proxy ratings (13.28 ± 15.89) than for the children's self‐reports (0.55 ± 9.44). Children experienced the greatest restrictions in the domains ‘treatment’ (21.88 ± 20.86), ‘family’ (20.83 ± 23.15) and ‘sport and school’ (12.50 ± 18.90). The parents reported the greatest HRQoL impairments of their children in the domains ‘family’ (22.92 ± 18.23), ‘view of yourself’ (15.63 ± 22.90), ‘treatment’ (14.06 ± 16.95) and ‘sport and school’ (14.06 ± 21.59). The parents’ total score in the retrospective survey on SHL‐FIX therapy using the Haemo‐QoL Index was 21.88 ± 16.34. In the 4–7‐year age group, the z‐standardized Pearson correlations between the children's and parents’ ratings ranged from *r* = –0.04 to *r* = 0.98. The highest agreement was found in the domains ‘feelings’ (*r* = 0.98; *p* < 0.001), ‘other people’ (*r* = 0.97; *p* < 0.001) and ‘physical health’ (*r* = 0.69; *p* = 0.0284).

The EQ‐5D‐Y‐3L resulted in a health profile with a mean ± SD score of 0.96 ± 0.08 for 8–16‐year‐olds. The mean EQ VAS score was 91.54 ± 7.18. Children aged 8–16 years experienced the greatest restrictions in the domains ‘friends’ (30.77 ± 24.62), ‘treatment’ (30.29 ± 23.22) and ‘family’ (M = 16.35 ± 12.38), according to the Haemo‐QoL SF II. The same domains were also identified as those with the greatest restrictions by their parents' proxy ratings. The total score for children and adolescents was 14.34 ± 5.94, while the parents' total score was 17.91 ± 7.56. In the retrospective survey on SHL‐FIX, the total score was 27.81 ± 15.76 for children and adolescents and 32.19 ± 11.60 for parents. Pearson correlations between children's self‐reports and parental proxy ratings in the 8–16 age group across the Haemo‐QoL domains ranged from *r*  = 0.21 to *r* = 0.81, indicating a low to very high degree of agreement. The highest concordance was found in the domain ‘treatment’ (r = 0.81; *p*  = 0.0001), followed by ‘friends’ (*r* = 0.66; *p* = 0.005) and ‘family’ (*r* = 0.57; *p* = 0.022).

The study participants aged 17 years and above had a mean ± SD EQ‐5D‐3L value of 0.94 ± 0.12 and an EQ VAS value of 88 ± 7.26. The Haem‐A‐QoL showed the greatest impairment in the ‘treatment’ (28.91 ± 10.32), ‘sport and leisure’ (27.50 ± 25.33) and ‘dealing with haemophilia’ (27.08 ± 10.46) domains, with a total score of 17.56 ± 5.63. The retrospective evaluation of SHL‐FIX in this age group revealed a total score of 17.50 ± 21.79.

## 4 Discussion

Licensing studies of extended‐half‐life FIX products for the treatment of HB promise lower infusion rates and higher trough levels with reduced bleeding rates [8, 9, 22, 23, 24]. The HAEMFIX study provides real‐world data on the transition from SHL products to EHL products, offering insights into changes in infusion rates, dosages, annual bleeding rates (ABRs) and HRQoL.

Compared with SHL‐FIX therapy (mean 145.0 infusions/year), treatment with EHL‐FIX resulted in a significantly lower number of infusions (mean 55.1 infusions/year). According to a 2017 survey, von Mackensen et al. reported that frequent infusions due to the short half‐life of SHL products were considered the main disadvantage of haemophilia therapy by patients [25]. The treatment regimen should be acceptable to the patient, considering their lifestyle and daily routine, while providing the highest level of bleed protection. The use of EHL products simplifies prophylaxis by reducing the frequency of infusions and may therefore increase adherence to treatment [26, 27, 28]. For patients and caregivers, in addition to safety and cost aspects, the reduction in weekly infusion frequency has a greater impact on therapy choice than minimal differences in efficacy between the preparations [29]. These findings indicate that EHL‐FIX treatment could have a beneficial effect on patients’ quality of life, as described in other studies [22, 24].

According to published data [22, 30, 31], in addition to reducing the infusion frequency, the weekly dosage of EHL‐FIX was also lower (mean value 56.4 IU/kg/week) than that of previously used SHL‐FIX (mean value 118.9 IU/kg/week). The reduction in both infusion frequency and weekly dosage led to a significantly lower annual factor consumption among study participants using EHL‐FIX (mean value 100,247.7 IU/year) than among those using SHL‐FIX (mean value 159,577.8 IU/year), confirming published data [22, 30, 31, 32, 33, 34, 35]. These findings suggest that using FIX products with extended half‐lives is cost‐effective and provides economic benefits in addition to individual advantages for patients. Higher factor consumption in older children and adolescents is due to increased body weight.

Despite the reduced dosage and infusion frequency due to the extended half‐life, it is possible to maintain higher FIX levels over a longer period. In our cohort, the estimated trough level of EHL‐FIX was 0.13 IU/mL after 7 days, which is comparable to previously published data and is in line with national and international treatment guidelines [22, 26]. The Wapps‐Hemo platform allows for individual precise predictions [15].

This study also revealed that, compared with SHL‐FIX, EHL‐FIX therapy resulted in a well‐controlled bleeding rate at a lower dosage. Upon switching to EHL‐FIX, the ABR significantly decreased, with a mean ABR of 2.85 under EHL‐FIX and 6.01 under SHL‐FIX therapy. Comparable studies have also demonstrated a decrease in bleeding rates when switching from SHL‐FIX to EHL‐FIX products. The decrease in the bleeding rate with increasing age may be attributed to extended half‐life of factor products and higher trough levels despite greater physical activity during childhood [22, 27, 30, 31, 32, 33, 34, 35, 36]. In this study, 72.4% of the participants treated with EHL‐FIX had an ABR of 0, which is consistent with previous studies [30, 34]. EHL‐FIX and SHL‐FIX both show high rates of patients with 0 bleeds per year (72.4% vs. 73.5%). Notably, EHL‐FIX led to fewer than two bleeding events a year in a greater percentage of patients (85.8%) than did SHL‐FIX (83.5%), suggesting better bleeding control. The slightly increased sABR in the EHL group might be explained by the number of younger patients in this group with preventive and documented factor substitutions in case of suspected bleeding without proven bleed. A decrease in ABR benefits both joint health and overall well‐being. This could be demonstrated in patients with a low HJHS (median 0) while on current EHL‐FIX therapy, with 95% of patients being free of joint morbidity and reporting a good HRQoL. Children in all age groups did not experience major limitations in their HRQoL during EHL‐FIX therapy. Haemophilia‐specific total scores indicated that parents rated the limitations slightly higher than did the children themselves. It is assumed that parents of chronically ill children underestimate their children's HRQoL [21, 37, 38]. A retrospective survey on HRQoL during SHL‐FIX treatment revealed improved HRQoL for the age groups 4–7 years and 8–16 years.

A limitation of this study is the small sample size, which is a common issue in rare disease research. Although data collection was performed in two of the largest paediatric haemophilia centres, the participation of other haemophilia care centres would provide additional information. Considering the rarity of the disease and the inclusion of only paediatric patients, the participation of 14% of paediatric patients registered with HB in the German haemophilia Registry in this cohort could be assumed to be a representative cohort [40]. The collected data are consistent with values from previous studies, also indicating the representativity of this study [22, 27, 30, 31, 34, 39]. Despite this representative number of patients, individual dosing regimens, age‐dependent pharmacokinetics, data derived from two different haemophilia centres and the relatively small cohort may have contributed to less homogenous data than expected. A larger overall cohort or age‐matched subcohorts might further enhance data homogeneity.

Using patient‐ or parent‐reported data might introduce an additional risk of reporting bias. Within the current electronic and paper‐based documentation systems, treatments can only be recorded as either prophylaxis or bleeding‐related. In younger or highly active children, suspected bleeding events following minor trauma—for example, a toddler bumping their head—are frequently documented as ‘bleeds’, even in the absence of clinical signs. Such precautionary treatment, administered solely to minimise a potential risk of bleeding, is therefore classified as treatment of a bleed, which may lead to an artificially increased documented bleeding rate. Furthermore, the retrospective evaluation of HRQoL under treatment with SHL‐FIX was not performed immediately after switching, but at study inclusion and could thus lead to a recall bias with poorer assessment of HRQoL among SHL treatment.

## 5 Conclusion

This study shows a great benefit of the use of EHL‐FIX products in patients with haemophilia B. Infusion frequency, factor consumption, ABR and, consequently, health care costs could be reduced while maintaining sufficiently high trough levels and improving health‐related quality of life. This study revealed no evidence of safety concerns with the use of EHL‐FIX products in real‐world dosing regimens.

## Author Contribution

M.O., J.L., C.B. and S.v.M. designed the study and contributed to the data analysis. M.O., J.L., C.B., S.H., J.H., S.J. and V.L. recruited patients, and L.K., J.F. and H.F. performed the statistical analysis. J.L. and M.O. drafted the initial manuscript. All the authors critically revised the manuscript and approved the final version of the manuscript.

## Funding

The authors have nothing to report.

## Ethics Statement

The study was approved by the ethics committee and data protection commissioner of the medical faculty of the Ludwig‐Maximilians‐University Munich, Germany, Project‐Nr. 22‐0680.

## Conflicts of Interest

The authors have nothing to report. Susan Halimeh: Speaker fees and travel grant from Bayer Healthcare GmbH, Takeda GmbH, Biotest AG, CSL Behring GmbH, Novo Nordisk Pharma GmbH, Octapharma GmbH, Pfizer Pharma, Roche Pharma AG; Swedish Orphan Biovitrum GmbH, Chugai Pharma Germany GmbH. Consultancy fees from Bayer Healthcare GmbH, Biotest AG, CSL Behring GmbH, Novo Nordisk Pharma GmbH, Octapharma GmbH, Chugai Pharma Germany GmbH, Swedish Orphan Biovitrum GmbH. Research Grants from Bayer Healthcare GmbH, Takeda GmbH, Biotest AG, Novo Nordisk Pharma GmbH, Octapharma GmbH. Sylvia von Mackensen: Consultancy for Pfizer, Roche and Spark; research funding from Biomarin, Swedish Orphan Biovitrum and Takeda; speakers bureau for Biomarin, Chugai, Kedrion, Pfizer and Takeda; membership of an entity's Board of Directors or advisory committee with Biomarin and Chugai/Roche. Lisa Kleinlein: Nothing to disclose. Juliet Fleischer: Nothing to disclose. Henri Funk: Nothing to disclose. Julia Hölz: Nothing to disclose. Johannes Holzapfel: speaker fees and travel grant from Biotest, Takeda, Roche, Swedish Orphan Biovitrum. Sabrina Juranek: speaker fees and travel grant from Biotest, CSL Behring, Pfizer, Swedish Orphan Biovitrum. Victoria Lieftüchter: Nothing to disclose. Christoph Bidlingmaier: travel grants, research grants and honoraria from Biotest, CSL Bering, Novo Nordisk, Roche, Swedish Orphan Biovitrum, Takeda. Martin Olivieri: received grants/research support from Bayer, Biotest, Takeda, CSL Behring, Octapharma, Pfizer, Shire, Roche and Swedish Orphan Biovitrum; consultancy and speaker fees from Bayer, Biotest, Novo Nordisk, Takeda, CSL Behring, Pfizer, Roche, Stago and Swedish Orphan Biovitrum.

## Data Availability

The data that support the findings of this study are available upon request from the HAEMFIX core team (please mail to: martin.olivieri@med.uni-muenchen.de). The data are not publicly available due to privacy or ethical restrictions.

## References

[1] P. H. Bolton‐Maggs and K. J. Pasi , “Haemophilias a and b,” The Lancet 361, no. 9371 (2003): 1801–1809. 10.1016/S0140-6736(03)13405-8 12781551

[2] A. Srivastava , A. K. Brewer , E. P. Mauser‐Bunschoten , et al., “Guidelines for the Management of Hemophilia,” Haemophilia 19, no. 1 (2013): e1–e47. 22776238 10.1111/j.1365-2516.2012.02909.x

[3] G. Ling , A. C. Nathwani , and E. G. Tuddenham , “Recent Advances in Developing Specific Therapies for Haemophilia,” British Journal of Haematology 181, no. 2 (2018): 161–172. 29359795 10.1111/bjh.15084

[4] A. Gater , T. A. Thomson , and M. Strandberg‐Larsen , “Haemophilia B: Impact on Patients and Economic Burden of Disease,” Thrombosis and Haemostasis 106, no. 09 (2011): 398–404. 21833450 10.1160/TH11-03-0193

[5] S. S. Acharya , “Exploration of the Pathogenesis of Haemophilic Joint Arthropathy: Understanding Implications for Optimal Clinical Management,” British Journal of Haematology 156, no. 1 (2012): 13–23. 22050780 10.1111/j.1365-2141.2011.08919.x

[6] M. Morfini , A. Coppola , M. Franchini , and G. Di Minno , “Clinical Use of Factor VIII and Factor IX Concentrates,” Blood Transfus 11, Suppl no. 4 (2013): s55–s63. 24333314 10.2450/2013.010s PMC3853992

[7] M. W. Skinner , “WFH: Closing the Global Gap–achieving Optimal Care,” Haemophilia 18 (2012): 1–12. 10.1111/j.1365-2516.2012.02822.x 22726075

[8] M. E. Mancuso , J. Oldenburg , L. Boggio , et al., “High Adherence to Prophylaxis Regimens in Haemophilia B Patients Receiving rIX‐FP: Evidence From Clinical Trials and Real‐world Practice,” Haemophilia 26, no. 4 (2020): 637–642. 32542961 10.1111/hae.14018 PMC7496492

[9] M. C. Ar , C. Balkan , and K. Kavaklı , “Extended Half‐life Coagulation Factors: A New Era in the Management of Hemophilia Patients,” Turkish Journal of Hematology 36, no. 3 (2019): 141. 31088040 10.4274/tjh.galenos.2019.2018.0393 PMC6682782

[10] E. Santagostino , “Transforming the Treatment for Hemophilia B Patients: Update on the Clinical Development of Recombinant Fusion Protein Linking Recombinant Coagulation Factor IX With Recombinant Albumin (rIX‐FP),” Thrombosis Research 141 (2016): S5–S8. 27288064 10.1016/S0049-3848(16)30415-7

[11] H. Fassel and C. McGuinn , “Haemophilia: Factoring in New Therapies,” British Journal of Haematology 194, no. 5 (2021): 835–850. 34322873 10.1111/bjh.17580

[12] M. Nazeef and J. P. Sheehan , “New Developments in the Management of Moderate‐to‐severe Hemophilia B,” Journal of Blood Medicine (2016): 27–38. 27099538 10.2147/JBM.S81520 PMC4824368

[13] K. J. Pasi , K. Fischer , M. Ragni , et al., “Long‐term Safety and Efficacy of Extended‐interval Prophylaxis With Recombinant Factor IX Fc Fusion Protein (rFIXFc) in Subjects With Haemophilia B,” Thrombosis and Haemostasis 117, no. 03 (2017): 508–518. 28004057 10.1160/TH16-05-0398

[14] M. E. Mancuso and E. Santagostino , “Outcome of Clinical Trials With New Extended Half‐life FVIII/IX Concentrates,” Journal of Clinical Medicine 6, no. 4 (2017): 39. 28350322 10.3390/jcm6040039 PMC5406771

[15] D. M. Hajducek , P. Chelle , C. Hermans , et al., “Development and Evaluation of the Population Pharmacokinetic Models for FVIII and FIX Concentrates of the WAPPS‐Hemo Project,” Haemophilia 26, no. 3 (2020): 384–400. 32281726 10.1111/hae.13977

[16] I. A. Kuijlaars , J. van der Net , B. M. Feldman , et al., “Evaluating International Haemophilia Joint Health Score (HJHS) Results Combined With Expert Opinion: Options for a Shorter HJHS,” Haemophilia 26, no. 6 (2020): 1072–1080. 33058441 10.1111/hae.14180 PMC7821332

[17] N. Wille , X. Badia , G. Bonsel , et al., “Development of the EQ‐5D‐Y: A Child‐friendly Version of the EQ‐5D,” Quality of Life Research 19, no. 6 (2010): 875–886. 20405245 10.1007/s11136-010-9648-y PMC2892611

[18] G. Balestroni and G. Bertolotti , “EuroQol‐5D (EQ‐5D): An Instrument for Measuring Quality of Life,” Monaldi Archives for Chest Disease 78, no. 3 (2012): 155–159. 23614330 10.4081/monaldi.2012.121

[19] S. von Mackensen and M. Bullinger , “Development and Testing of an Instrument to Assess the Quality of Life of Children With Haemophilia in Europe (Haemo‐QoL),” Haemophilia 10, Suppl no. 1 (2004): 17–25. 10.1111/j.1355-0691.2004.00875.x 14987245

[20] S. von Mackensen and A. Gringeri , “Quality of Life in Hemophilia,” Textbook of Hemophilia (2014): 478–488. 10.1111/j.1365-2516.2008.01709.x 18510517

[21] E. Pollak , H. Mühlan , S. VONM , and M. Bullinger , “The Haemo‐QoL Index: Developing a Short Measure for Health‐related Quality of Life Assessment in Children and Adolescents With Haemophilia,” Haemophilia 12, no. 4 (2006): 384–392. 16834738 10.1111/j.1365-2516.2006.01292.x

[22] M. Escobar , M. E. Mancuso , C. Hermans , et al., “IDELVION: A Comprehensive Review of Clinical Trial and Real‐world Data,” Journal of Clinical Medicine 11, no. 4 (2022): 1071. 35207344 10.3390/jcm11041071 PMC8875492

[23] C. Marlière , L. Maindiaux , C. Lambert , and C. Hermans , “EHL‐FIX in Haemophilia B Carriers With FIX Deficiency,” Haemophilia 26 (2020): e38. 31840356 10.1111/hae.13906

[24] J. N. Mahlangu , “Updates in Clinical Trial Data of Extended Half‐life Recombinant Factor IX Products for the Treatment of Haemophilia B,” Therapeutic Advances in Hematology 9, no. 11 (2018): 335–346. 30364483 10.1177/2040620718802606 PMC6196631

[25] S. Von Mackensen , W. Kalnins , J. Krucker , et al., “Haemophilia Patients' unmet Needs and Their Expectations of the New Extended Half‐life Factor Concentrates,” Haemophilia 23, no. 4 (2017): 566–574. 28370896 10.1111/hae.13221

[26] A. Rampotas , M. J. Desborough , S. Raza‐Burton , et al., “A Single Centre Retrospective Study of Low Dose Prophylaxis With Extended Half‐life Factor IX for Severe Haemophilia B,” Haemophilia 26, no. 2 (2020): 278–281. 32083769 10.1111/hae.13936

[27] M. O'Donovan , E. Quinn , K. Johnston , E. Singleton , J. Benson , and B. O'Mahony , “Recombinant Factor IX‐Fc Fusion Protein in Severe Hemophilia B: Patient‐reported Outcomes and Health‐related Quality of Life,” Research and Practice in Thrombosis and Haemostasis 5, no. 7 (2021): e12602. 34667923 10.1002/rth2.12602 PMC8505226

[28] C. D. Thornburg and N. A. Duncan , “Treatment Adherence in Hemophilia,” Patient Prefer Adherence 11 (2017): 1677–1686. 29033555 10.2147/PPA.S139851 PMC5630068

[29] R. Furlan , S. Krishnan , and J. Vietri , “Patient and Parent Preferences for Characteristics of Prophylactic Treatment in Hemophilia,” Patient Preference and Adherence 9 (2015): 1687–1694. 26648701 10.2147/PPA.S92520 PMC4664548

[30] J. Oldenburg , S. Yan , G. Maro , G. Krishnarajah , and A. Tiede , “Assessing Bleeding Rates, Related Clinical Impact and Factor Utilization in German Hemophilia B Patients Treated With Extended Half‐life rIX‐FP Compared to Prior Drug Therapy,” Current Medical Research and Opinion 36, no. 1 (2020): 9–15. 31469321 10.1080/03007995.2019.1662675

[31] C. Hermans , R. Marino , C. Lambert , et al., “Real‐world Utilisation and Bleed Rates in Patients With Haemophilia B Who Switched to Recombinant Factor IX Fusion Protein (rIX‐FP): A Retrospective International Analysis,” Advances in Therapy 37 (2020): 2988–2998. 32333327 10.1007/s12325-020-01300-6 PMC7467451

[32] T. Burke , S. Asghar , J. O'Hara , M. Chuang , E. K. Sawyer , and N. Li , “Clinical, Humanistic, and Economic Burden of Severe Haemophilia B in Adults Receiving Factor IX Prophylaxis: Findings From the CHESS II Real‐world Burden of Illness Study in Europe,” Orphanet Journal of Rare Diseases 16 (2021): 1–9. 34930388 10.1186/s13023-021-02152-1 PMC8691083

[33] Y. Brennan , S. Parikh , S. McRae , and H. Tran , “The Australian Experience With Switching to Extended Half‐life Factor VIII and IX Concentrates: On Behalf of the Australian Haemophilia Centre Directors' Organisation,” Haemophilia 26, no. 3 (2020): 529–535. 32243027 10.1111/hae.13970

[34] S. Pasca and E. Zanon , “Albumin‐Fusion Recombinant FIX in the Management of People With Hemophilia B: An Evidence‐Based Review,” Drug Design, Development and Therapy 16 (2022): 3109–3116. 36132333 10.2147/DDDT.S236788 PMC9484766

[35] C. Wang and G. Young , “Clinical Use of Recombinant Factor VIII Fc and Recombinant Factor IX Fc in Patients With Haemophilia A and B,” Haemophilia 24, no. 3 (2018): 414–419. 29405496 10.1111/hae.13432

[36] A. Chhabra , D. Spurden , P. F. Fogarty , et al., “Real‐World Outcomes Associated With Standard Half‐Life and Extended Half‐Life Factor Replacement Products for Treatment of Haemophilia A and B,” Blood Coagulation & Fibrinolysis 31, no. 3 (2020): 186. 32271314 10.1097/MBC.0000000000000885 PMC7195855

[37] C. Eiser and J. L. Berrenberg , “Assessing the Impact of Chronic Disease on the Relationship Between Parents and Their Adolescents,” Journal of Psychosomatic Research 39 (1995): 109–114. 7595868 10.1016/0022-3999(94)00099-q

[38] C. Eiser and R. Morse , “Quality‐of‐Life Measures in Chronic Diseases of Childhood,” Health Technology Assessment (Winchester, England) 5, no. 4 (2001): 1–157. 10.3310/hta5040 11262421

[39] F. Peyvandi , I. Garagiola , M. Boscarino , A. Ryan , C. Hermans , and M. Makris , “Real‐Life Experience in Switching to New Extended Half‐Life Products at European Haemophilia Centres,” Haemophilia 25, no. 6 (2019): 946–952. 31418967 10.1111/hae.13834

[40] https://www.pei.de/SharedDocs/Downloads/DE/regulation/meldung/dhr‐deutsches‐haemophilieregister/dhr‐jahresbericht‐2021.pdf?__blob=publicationFile&v=4 (23.01.2025).

